# Early amygdala and ERC atrophy linked to 3D reconstruction of rostral neurofibrillary tau tangle pathology in Alzheimer’s disease^☆^

**DOI:** 10.1016/j.nicl.2023.103374

**Published:** 2023-03-15

**Authors:** Kaitlin M. Stouffer, Claire Chen, Sue Kulason, Eileen Xu, Menno P. Witter, Can Ceritoglu, Marilyn S. Albert, Susumu Mori, Juan Troncoso, Daniel J. Tward, Michael I. Miller, for the Alzheimer’s Disease Neuroimaging Initiative

**Affiliations:** a Department of Biomedical Engineering, Johns Hopkins University, 3400 N Charles St, Baltimore 21218, MD, USA; b Departments of Computational Medicine and Neurology, University of California, Los Angeles, UCLA Brain Mapping Center, 660 Charles E. Young Drive South, Los Angeles 90095, CA, USA; c Departments of Neurology, Johns Hopkins School of Medicine, 733 N Broadway, Baltimore 21205, MD, USA; d Department of Pathology, Johns Hopkins School of Medicine, 733 N Broadway, Baltimore 21205, MD, USA; e Department of Radiology, Johns Hopkins School of Medicine, 733 N Broadway, Baltimore 21205, MD, USA; f Kavli Institute for Systems Neuroscience, Norwegian University of Science and Technology, 7491 Trondheim, Norway

**Keywords:** Alzheimer’s disease, MRI biomarkers, Amygdala atrophy, Digital pathology

## Abstract

Previous research has emphasized the unique impact of Alzheimer’s Disease (AD) pathology on the medial temporal lobe (MTL), a reflection that tau pathology is particularly striking in the entorhinal and transentorhinal cortex (ERC, TEC) early in the course of disease. However, other brain regions are affected by AD pathology during its early phases. Here, we use longitudinal diffeomorphometry to measure the atrophy rate from MRI of the amygdala compared with that in the ERC and TEC in cognitively unimpaired (CU) controls, CU individuals who progressed to mild cognitive impairment (MCI), and individuals with MCI who progressed to dementia of the AD type (DAT), using a dataset from the Alzheimer’s Disease Neuroimaging Initiative (ADNI). Our results show significantly higher atrophy rates of the amygdala in both groups of ‘converters’ (CU→MCI, MCI→DAT) compared to controls, with rates of volume loss comparable to rates of thickness loss in the ERC and TEC. We localize atrophy within the amygdala within each of these groups using fixed effects modeling. Controlling for the familywise error rate highlights the medial regions of the amygdala as those with significantly higher atrophy in both groups of converters than in controls. Using our recently developed method, referred to as Projective LDDMM, we map measures of neurofibrillary tau tangles (NFTs) from digital pathology to MRI atlases and reconstruct dense 3D spatial distributions of NFT density within regions of the MTL. The distribution of NFTs is consistent with the spatial distribution of MR measured atrophy rates, revealing high densities (and atrophy) in the amygdala (particularly medial), ERC, and rostral third of the MTL. The similarity of the location of NFTs in AD and shape changes in a well-defined clinical population suggests that amygdalar atrophy rate, as measured through MRI may be a viable biomarker for AD.

## Highlights

• Amygdala atrophy rate estimated from MRIs prior to onset of MCI and AD dementia.
• Comparable atrophy rates in amygdala and ERC prior to onset of MCI and AD dementia.
• 3D tau tangle (NFT) distribution reconstructed from 2D histology in 2 advanced AD cases.
• NFTs highest in the rostral medial temporal lobe, including amygdala and ERC.
• MRI-based amygdala atrophy largest in areas with highest reconstructed NFT density.

## 1 Introduction

Alzheimer’s disease (AD) is the leading cause of dementia worldwide (Association, 2021). Diagnosis and characterization of AD in its *early* stages remain key challenges, as existing technologies limit the identification of the neuropathological patterns thought to emerge years before symptom onset (Sperling et al., 2011, Nauen and Troncoso, 2021, Villemagne et al., 2018). In clinical practice, AD is typically first characterized by progressive clinical changes in memory and behavior, and subsequently through imaging changes that indirectly reflect AD neuropathology (i.e. misfolded proteins, tau and amyloid-Beta (Aβ)) (Braak and Braak, 1991, Braak et al., 2006, Hyman et al., 2012). Efforts to identify and understand the spatiotemporal profile of AD in its early stages have centered on these biomarkers (Jack et al., 2018)–measures that indirectly reflect the underlying pathology, which are obtainable over the course of disease. Of the methods used, neuroimaging has emerged as a prominent player with the ability to localize pathology non-invasively (e.g. tau/amyloid positron emission tomography (PET)) (Zetterberg and Bendlin, 2021, Young et al., 2020), and with proposed surrogates such as shape diffeomorphometric markers (e.g. magnetic resonance imaging (MRI)) (Younes et al., 2014, Younes et al., 2019, Kulason et al., 2019). While these imaging measures have shown consistency with progression of clinical manifestations and with Braak staging (Braak and Braak, 1991, Braak et al., 2006, Mattsson et al., 2019, Therriault et al., 2022), accurate rendering of the 3D spatiotemporal profile of tau and Aβ at the micron scale has not been achieved (Zetterberg and Bendlin, 2021, Young et al., 2020). The principal challenge has been integrating the 2D sparse measurements of histology, which are direct measures of disease, with the MRI 3D markers, which are at much lower in-plane resolution. Consequently, these imaging measures have tended to emphasize MTL regions, including the entorhinal cortex (ERC) and hippocampus. These measurements, have not, however, been linked directly to micron level patterns of tau and Aβ pathology–the hallmark findings of AD.

The amygdala is one region that has undergone relatively limited study in AD (Wang et al., 2021) compared to its adjoining regions of the ERC and hippocampus in the medial temporal lobe (MTL) highlighted by Braak and Braak (1991). Neuropathological and connectivity-based studies suggest a role for the amygdala in neurodegenerative diseases such as Argyrophilic Grain Disease (AGD) (Ferrer et al., 2008), Lewy Body dementia (Popescu et al., 2004), and AD (Nelson et al., 2018, Arnold et al., 1991) with the observed inclusion of misfolded proteins in the amygdala and the its strong connectivity patterns to areas of the hippocampus, basal ganglia, and basal forebrain (Nelson et al., 2018). Imaging studies from our group and others have observed cross-sectional differences in amygdala volume between cognitively unimpaired (CU) individuals, those with mild cognitive impairment (MCI), mild AD dementia patients and later stage AD patients (Miller et al., 2015a, Miller et al., 2015b, Kulason et al., 2019, Ortner et al., 2016). These shape differences complement recent findings in tau PET illustrating cross-sectional differences in tau load in areas of the rostral MTL including ERC, transentorhinal cortex (TEC), and amygdala, in early stages of AD (Berron et al., 2021). Two studies have also shown correlation between neuropsychiatric symptom severity and amygdalar atrophy in AD (Poulin et al., 2011, Wang et al., 2021). These findings, together with emerging evidence of the neuropsychiatric syndromic complex known as Mild Behavioral Impairment (MBI) amongst individuals prior to the onset of dementia (Johansson et al., 2021, Matuskova et al., 2021) suggest amygdalar changes early in the disease course may be related to the emotional and behavioral syndrome of MBI. Consequently, assessment of amygdala atrophy via modes such as MRI holds promise as a biomarker for early diagnosis and management of AD. However, the exact timing and rate at which this atrophy occurs have not been well established as in other regions such as the ERC (Kulason et al., 2020), but which is necessary for development of an appropriate biomarker.

To assess these rates, we compute both ERC thickness and amygdalar volume over time in MRI scans from a subset of the Alzheimer’s Disease Neuroimaging Intiative (ADNI) subjects in one of three categories: (1) CU subjects at baseline who remain CU at follow-up, (2) CU subjects at baseline who progress to MCI, and (3) subjects with a diagnosis of MCI at baseline who progress to dementia of the Alzheimer’s type (DAT) on follow-up. These groups are designated with the abbreviations CU→CU, CU→MCI, and MCI→DAT, respectively. Using an established approach of longitudinal diffeomorphometry (Tward and Miller, 2017), we estimate percent atrophy per year in each structure for each subject by fitting each subject’s series of scans to a smooth trajectory to extract signal from noise. We additionally use the mappings estimated through longitudinal diffeomorphometry to compute average atrophy rate across each group of subjects at each location on a common amygdala hypertemplate. We use linear fixed effects modeling and control for the familywise error rate (FWER) to deduce which areas of the amygdala show significant differences in atrophy rate across these three groups.

We subsequently link both these ERC and amygdalar atrophy rates as MRI-based measures at a millimeter scale to patterns of AD pathology. We recently developed a method, referred to as Projective LDDMM (Large Deformation Diffeomorphic Metric Mapping), which facilitates the integration of multi-scale, multi-modal data into a single coordinate system (Stouffer et al., 2022). Here, we use this approach to reconstruct 3D distributions of neuropathology within the MTL of advanced cases of AD, with a focus on comparing relative load of pathology between the ERC and amygdala and other regions of the hippocampus and within particularly the amygdala as relates to the areas of significant atrophy seen in CU→MCI and MCI→DAT groups. As tau has exhibited stronger predisposition over Aβ for segregating to particular brain regions (ERC, Cornu Ammonis 1 (CA1), subiculum) and layers (superficial) of cortex in AD (Braak and Braak, 1991), we use machine-learning based methods to detect neurofibrillary tangles (NFTs) from histological images. We quantify these detections in the space of histology images as measures of NFT density (per cross sectional tissue area) and carry these measures to the space of high field 11T 3D MRI and the Mai Paxinos Atlas (Mai et al., 2008) using the transformations we estimate via Projective LDDMM. Using manual delineations on high field ex vivo MRI of subregions in the MTL and within both the amygdala and ERC, we quantify relative pathology within and among substructures for further corroboration of manifest atrophy rate in clinical populations progressing to MCI and early AD.

## 2 Material and methods

### 2.1 Subject selection and image processing

#### 2.1.1 Longitudinal data selection

The subjects and scans analyzed in this study were selected from the ADNI database (adni.loni.usc.edu) according to the criteria used in our previous studies (Kulason et al., 2019, Kulason et al., 2020), where groups are identified based on cognitive status. The ADNI was launched in 2003 as a public–private partnership, led by Principal Investigator Michael W. Weiner, MD. The primary goal of ADNI has been to test whether serial MRI, PET, other biological markers, and clinical and neuropsychological assessment can be combined to measure the progression of MCI and early AD.

**Fig. 1:**
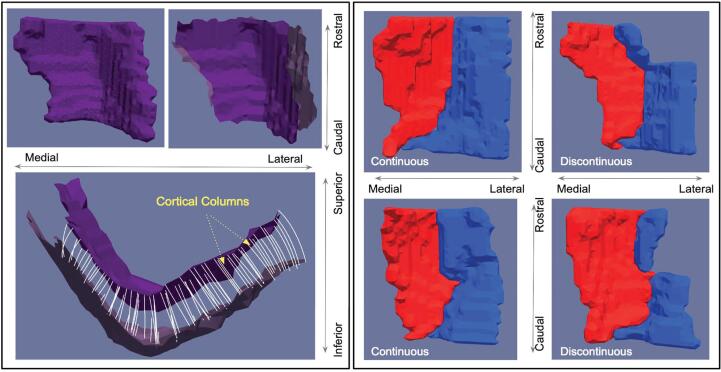
3D surfaces of ERC + TEC geometry rendered from manual segmentations of 3T MR scans. Left panel shows surface with corresponding cortical columns (bottom) estimated between inner and outer layers (top right) for estimation of thickness. Right panel shows ERC (red) and TEC (blue) geometry for individuals with anteriorly continuous CS (left) and those without (right).

In this current study, all subjects in all groups were required to have at least three 3T MR scans for longitudinal comparison. To ensure accurate and consistent segmentation of the entorhinal and transentorhinal regions across subjects with varying types of collateral sulcus (CS), all subjects were required to have an anteriorly continuous CS, as detailed in Kulason et al. (2020). The differences in the resulting geometries of these regions between subjects with and without an anteriorly continuous CS are demonstrated in Fig. 1, highlighting the complexity and consequently the ambiguity introduced in maintaining a consistent segmentation protocol across subjects. This restriction to subjects only with an anteriorly continuous CS enabled us to reduce any confounding factors that may have arisen in the smoothing process when mapping a template geometry of one type to a target geometry of potentially another. Instead, we mapped the same single template to each subject’s time series for longitudinal smoothing as the subset of subjects with an anteriorly continuous CS maintained a more consistent geometry in the entorhinal region than the group as a whole.

Three subgroups of this subset were selected based on diagnostic diagnosis at baseline and follow-up: (1) CU subjects at baseline and follow-up, abbreviated as “CU→CU”; (2) subjects unimpaired at baseline and diagnosed with MCI on follow-up, abbreviated as “CU→MCI” (NB: based on ADNI procedures, the subjects with normal cognition were labeled (CN), had a CDR score (Hughes et al., 1982) of 0, and normal range performance on the Logical Memory Subtest of the Wechsler Memory Scale (Wechsler, 1945) according to education adjusted norms); (3) subjects with a diagnosis of MCI at baseline who subsequently progressed to a diagnosis of dementia of the Alzheimer’s type at follow-up, abbreviated as “MCI→DAT”. Table 1 summarizes the demographics of each of these three groups. Additionally, all subjects in the CU→CU group carried Aβ negative status, as measured by cerebrospinal fluid (CSF) Aβ1-42 with a cutoff of 192pg/mL, as determined by the ADNI Biospecimen core. In contrast, those in the MCI→DAT group had Aβ positive status at baseline. Aβ status varied in the CU→MCI group with 7 subjects with confirmed Aβ positive status at baseline, 4 confirmed with Aβ negative status at baseline, and 5 without recorded CSF measures. Note that the accelerated scans of the ADNI 3 protocol were not included in this study.

**Table 1:** Demographics of three subgroups of ADNI dataset. Statistics reported as mean ± standard deviation where appropriate. Time of diagnosis reported as onset of MCI in CU→MCI group and onset of dementia in MCI→DAT group.

**Category**	**CU**→**CU**	**CU**→**MCI**	**MCI**→**DAT**
Sample Size	33	16	18
Baseline Age (years)	72.3 ± 5.5	74.6 ± 5.2	72.8 ± 6.6
Sex (% female)	45.5	75.0	61.1
# of scans	3.9 ± 0.2	4.0 ± 0.6	3.8 ± 0.4
Scan period (years)	2.3 ± 0.7	1.9 ± 0.9	1.8 ± 0.4
Time of Diagnosis	—	4.1 ± 2.6	2.7 ± 1.8
(years since baseline)			

#### 2.1.2 Ex vivo specimen preparation and imaging

Brain tissue samples for ex vivo analysis were prepared from two cases of advanced AD by the Johns Hopkins Brain Resource Center. One hemisphere from each sample was reserved for neuropathological staging and diagnosis. The two samples examined in this study had clinical diagnoses of advanced AD and Braak stage VI pathology (Braak et al., 2006), indicative of high levels of tau pathology throughout the MTL. Detailed demographics and pathological staging of each sample analyzed are summarized in Table 2.

**Table 2:** Donor demographics and pathological staging for ex vivo brain samples.

**Category**	**Subject 1**	**Subject 2**
Age	93	87
Sex	M	F
Clinical Diagnosis	Dementia	Dementia
Braak NFT stage	VI/VI	VI/VI
CERAD Neuritic Plaque Score (Mirra et al., 1991)	B	B
Thal Aβ Plaque Score (Thal et al., 2002)	5	5
TDP-43	−	+
Pathologic Diagnosis (Hyman et al., 2012)	High level AD pathologic change; multiple cerebral infarcts	High level AD pathologic change

The other hemisphere was immersion fixed in 10% buffered formalin prior to dissection. A portion of the MTL extending from the temporal pole to the hippocampal tail was excised in 3 contiguous blocks of tissue, sized 20–30 mm in height and width, and 15 mm rostral-caudal. Each block was imaged with an 11T MR scanner at 0.125 mm isotropic resolution by the Mori lab at Johns Hopkins. Subsequently, the blocks were serially sliced into 10 micron thick sections, with sets of 5–6 sections taken approximately every 1 mm. Each block yielded between 7 and 15 sets of sections. The first section from each set was stained with PHF-1 for tau tangle detection. Remaining sections in each set were reserved for calibration of NFT detections between brain samples (see Section 2.4.2) or Nissl staining for confirming 3D MRI segmentations (see Section 2.1.3). All stained sections were digitized at 2 micron resolution.

#### 2.1.3 Regional segmentations

Manual segmentations of MTL subregions were delineated on clinical (3T) and ex vivo (11T) MRI by a team of two individuals (CC and EX) guided by a neuroanatomist (MW). All segmentations were drawn with Seg3D version 1.13.0 (CIBC, 2016). Prior to segmentation, brightness and contrast on the MRI was adjusted for better visualization. A brush size of 1 voxel was selected for precision. Segmentation was primarily done on the coronal plane, while the axial and sagittal plane were used to clarify borders.

In 3T MR scans, ERC and TEC were delineated following the protocol used previously (Kulason et al., 2019, Kulason et al., 2020, Tward and Miller, 2017). Visible anatomical landmarks were defined to match described cytoarchitectonic borders (Insausti et al., 1998, Ding and Van Hoesen, 2010). Anteriorly, the ERC and TEC extended 4 mm beyond the hippocampal head. Posteriorly, the ERC and TEC extended 2 mm beyond the gyrus intralimbicus (GI) (Insausti et al., 1998). Medially, the ERC extended to the edge of the visible gray/white matter boundary. Laterally, the TEC extended to the deepest portion of the collateral sulcus. Finally, the border between lateral ERC and medial TEC was defined at the medial bank of the collateral sulcus, depending on the depths of the sulcus in each individual following a previously described protocol (Insausti et al., 1998). In this work, both ERC and TEC were combined into a single entorhinal region as a point of single comparison to the atrophy rates measured in the amygdala in these same subjects.

The amygdala was segmented manually in all individuals in coronal slices. Segmentation was done slice by slice from posterior to anterior following a protocol based on intensity contrast and comparison with anatomical landmarks identified for the hippocampus. Posteriorly, the region of dark contrast superior to the hippocampus, representing the inferior horn of the lateral ventricle, was used to delineate the anterior hippocampus from the posterior start of the amygdala. At that level, the first set of voxels marked as amygdala were those exhibiting an intermediate gray level of intensity, rather than white matter contrast as appears posteriorly. Over its antero-posterior extent, the amygdala was defined as an area with even intensity contrast that increased in size anteriorly. The anterior border was demarcated by the disappearance of contrast, which occurred, on average, 11.8 mm ± 2.0 mm from the marked posterior border. For consistency, a space of one voxel width is left unsegmented between the amygdala and ERC and TEC.

MRI scans from each subject at each time point were all segmented individually to maximize the accuracy of each segmentation to each scan. To reduce inter-rater variance, one individual (CC) segmented only amygdalas whereas the other (EX) segmented only ERC + TECs. To reduce intra-rater variance, both individuals underwent one month of training in which they segmented similar structures across different datasets of 3T MRIs prior to beginning work on this select ADNI subset. Measures of intra-rater variance for the scans included in the present study were assessed by comparing the Dice overlap score between replicate segmentation masks of the same structure in the same MR scan, made by the same individual at different points in time. Supplementary Table A.1 shows the distribution of overlap scores in a subset of 2 scans from 6 subjects from the ADNI data set. Each scan’s amygdala was segmented three times by the same individual and overlaps were computed between the first and second times and second and third times to assess consistency. Average overlap between two replicated segmentations was 0.87±0.04, with 4-11 days between segmentation attempts. See Supplementary Section Appendix A for details.

In ex vivo 11T scans, MTL subregions included amygdala, ERC, CA fields, subiculum, presubiculum, parasubiculum, and dentate gyrus. Segmentations were drawn following manual alignment of individual block MRIs into a single MRI using an in-house rigid alignment tool. Boundaries for each region were defined based on intensity differences and published MR segmentations (Ding et al., 2017, Wisse et al., 2012, Yushkevich et al., 2009). Where available, Nissl-stained histology sections were overlaid with MRI for validation of the intensity-based protocol. For assessment of NFT density distribution within the amygdala, the amygdala segmentation in a control brain sample was subdivided into five individual regions based on visible intensity differences in 11T images, combined with anatomical markers and previously published delineations (Amunts et al., 2006, Saygin et al., 2017, Olga et al., 2017). Regions include basolateral (BLA), basomedial (BMA), combined cortical, medial, and central (CMA), and lateral (LA) nuclei and periamygdaloid area (PA).

For evaluating accuracy of alignment between digital pathology and MRI, as done previously (Stouffer et al., 2022), all digital pathology images from a single brain sample were segmented into corresponding MTL subregions. These segmentations were drawn on histology images that had been downsampled by four times to a resolution of 32 microns in both the x and y directions. The protocol for segmentation of 2D images used previously published delineations of MTL subregions based on cytoarchitectonic characteristics (Amunts et al., 2006, Olga et al., 2017, Ding, 2013, Ding and Hoesen, 2015, Insausti et al., 1995, Insausti et al., 2019).

### 2.2 Morphometric measuring of atrophy rate

#### 2.2.1 Smoothing via longitudinal 3D diffeomorphometry

We use geodesic shooting as described in Tward and Miller, 2017, Thomas Fletcher, 2013 to estimate a smooth atrophy rate for each individual’s time series by shooting a hypertemplate through each time-series. This yields a smooth evolution of each patient’s starting geometry effectively acting as a filter, eliminating variance in volumetric calculations due to both imaging parameters such as head placement, specific machine, etc. and intra-rater variability in manual segmentation of the given structure. As in Large Deformation Diffeomorphic Metric Mapping (LDDMM) (Beg et al., 2005), we generate a flow, $\varphi_{t}$, from a smooth time-varying velocity field acting as the control $v_{t}$ satisfying $\overset{˙}{\varphi_{t}}=v_{t}\circ \varphi_{t}$, with initial condition $\varphi_{0}$. Shooting implies the geodesic flows are defined entirely by their initial momentum at time t=0 (Miller et al., 2006), which is denoted here as $p_{0}$:

(1a) $$\dot{p_{t}}=-{\mathrm{Dv}}_{t}^{T}\circ \varphi_{t}p_{t},\;\mathrm{initial}\;\mathrm{condition}\;p_{0},$$

determining the control and flow:

(1b) $$\left\{\begin{array}{c} v_{t}(\cdot )=\sum_{i}K(\cdot ,\varphi_{t}(x_{i}^{S}))p_{i,t}\\ {\dot{\varphi }}_{t}=v_{t}\circ \varphi_{t},\;\mathrm{initial}\;\mathrm{condition}\;\varphi_{0} \end{array}\right),$$

where *D* denotes the Jacobian over space and K(·,·) the reproducing kernel in the Hilbert space of smooth vector fields. The kernel determines the norm for the momentum $\|p{\|}^{2}=\sum_{i,j}p_{i}^{T}K(x_{i},x_{j})\;p_{j}$.

The connected subvolumes of amygdala and combined ERC and TEC for each subject are represented as a time-series, $S_{t_{1}},\ldots ,S_{t_{k}}$ with the simulation time interval normalized as $0<t_{1}<\ldots <t_{k}<1$. Each $S_{t_{i}}$ is a surface triangulation of vertices $S={\left\{x_{i}^{S}\in R^{3}\right\}}_{i=1:V}$constructed from the segmentation masks for each scan (see Section 2.2.2 for details). Geodesic shooting solves for the flow of the population template $S^{\mathrm{temp}}$ through the time-series by moving the points in each surface under the diffeomorphism according to $\varphi \cdot S={\left\{\varphi (x_{i}^{S})\in R^{3}\right\}}_{i=1:V}$. We insert the template into the time series at the optimized time $t^{*}$, denoting the template surface here as $S_{t^{*}}^{\mathrm{temp}}$. Using geodesic shooting, we parameterize the flows with the initial momentum, ρ at $t^{*}$ and flow the template backwards $\varphi_{t}^{\rho -},t<t^{*}$ fitting the early surfaces $S_{t_{i}},t_{i}<t^{*}$, and forwards $\varphi_{t}^{\rho +},t⩾t^{*}$ fitting the later surfaces $S_{t_{i}},t_{i}⩾t^{*}$, with the flows given according to:

(2) $$\left\{\begin{array}{c} {\overset{˙}{\varphi }}_{t}^{\rho +}=v_{t}^{\rho +}\circ \varphi_{t}^{\rho +},[t^{*}⩽t⩽1]\\ {\overset{˙}{\varphi }}_{t}^{\rho -}=v_{t}^{\rho -}\circ \varphi_{t}^{\rho -},[0⩽t<t^{*}] \end{array}\right),\;\varphi_{t^{*}}^{\rho +}=\varphi_{t^{*}}^{\rho -}=\mathrm{Id}\text{.}$$

The variational problem thus optimizes on the single initial condition $p_{t^{*}}^{-}=p_{t^{*}}^{+}=\rho$, with (1a) and (1b) satisfied for $v_{t}^{\rho -},v_{t}^{\rho +},\varphi_{t}^{\rho +},\varphi_{t}^{\rho -}$:

(3) $$\underset{\rho }{\inf}\|\rho {\|}^{2}+\underset{t_{i}<t^{*}}{\sum }\|S_{t_{i}}-\varphi_{t_{i}}^{\rho -}\cdot S_{t^{*}}^{\mathrm{temp}}{\|}_{W^{*}}^{2}+\underset{t_{i}⩾t^{*}}{\sum }\|S_{t_{i}}-\varphi_{t_{i}}^{\rho +}\cdot S_{t^{*}}^{\mathrm{temp}}{\|}_{W^{*}}^{2}\text{.}$$

At the interface, $v_{t^{*}}^{\rho -}=v_{t^{*}}^{\rho +}=K\rho$ (Tward and Miller, 2017). For each subject’s time-series we also optimize over the insertion point $t^{*}$ and the flow of the fixed common template onto the unique target $S_{t^{*}}^{\mathrm{temp}}$. We do this by jointly optimizing (3) with the flow of the common template onto the target $S_{t^{*}}^{\mathrm{temp}}$.

#### 2.2.2 Amygdala volume measurements

3D surface mesh renderings of the amygdala were generated for each subject at each time point using restricted Delaunay triangulation (Cheng et al., 2013). As described in the previous section, we define each surface *S* as a collection of vertices, $S={\left\{x_{i}^{S}\in R^{3}\right\}}_{i\in I}$, together with a family of 3-tuples, $C=(c=(c_{1},c_{2},c_{3})\in I^{3})$ of indexes such that each cell is defined as $\gamma_{c}(S)=\left\{\sum_{i=1}^{3}a_{i}x_{c_{i}},\sum_{i}a_{i}=1,a_{i}⩾0\right\}$, with non-empty interior, with positive orientation, area $\left|\gamma_{c}(S)|≔\frac{1}{2}|(x_{c_{2}}-x_{c_{1}})\times (x_{c_{3}}-x_{c_{1}})\right|$ and cell centers $m_{c}(S)=\frac{1}{3}(x_{c_{1}}+x_{c_{2}}+x_{c_{3}})$. Amygdalar volumes were computed from triangulated surfaces by summing the signed individual volumes of tetrahedrons formed by each cell together with the origin $V=\sum_{c\in C}\frac{1}{6}(x_{c_{1}}\times x_{c_{2}}\cdot x_{c_{3}})$.

**Fig. 2:**
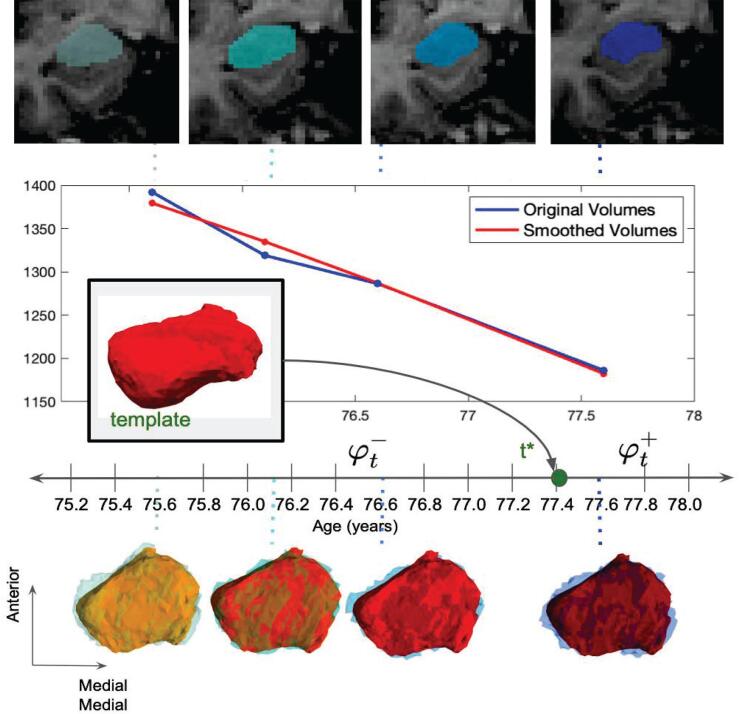
Smoothed flow of amygdalar surface for subject in CU →MCI group. Coronal slice of MRI at each time point shown with amygdala segmentation (top), from which original amygdalar surfaces are generated. Measured volumes of original surfaces plotted in blue (middle). Longitudinal shooting estimates flow from template to target, entry point into target series ($t^{*}$), and flow through time series backward and forward ($\varphi_{t}^{-},\varphi_{t}^{+}$). Flowed template surface sampled at each of four time points shown in red overlaying original subject surfaces in blue (bottom). Measured volumes of estimated smoothed surfaces plotted in red (middle).

Atrophy rate per subject was measured in the amygdala as change in volume per time (mm^3^/year). Rates of change were estimated per subject for a subject’s entire time series via least squares fitting for each of the three groups. For comparison between subjects, rates were computed as a percent change in volume from the starting measure in each series of time points. The hyper-template surface mesh from a high field ex vivo MRI (0.125 mm resolution) of a 27 year old male without AD pathology allowing us to bring the clinical population into register with the pathology specimen. This sample was prepared according to the protocol described in Section 2.1.2, and both regional and subregional delineations of the amygdala were drawn on this MRI following the protocol described in Section 2.1.3. This process is summarized in Fig. 2, where amygdala surfaces for one subject at each of four time points are shown in blue, overlaid with corresponding surfaces of the template’s flow sampled at these same time points.

#### 2.2.3 ERC plus TEC thickness measurements

3D surface mesh of the combined ERC and TEC were generated for each subject at each time point as above. Vertex-wise thickness of combined ERC and TEC surfaces was computed following the established approach of Ratnanather et al. (2019), as used in our previous work (Kulason et al., 2019, Kulason et al., 2020). Surfaces were cut into an outer (pial) surface and inner (gray/white matter boundary) surface using an in-house manual tool. Diffeomorphic transformations of the inner surface to the outer surface were estimated with LDDMM with the added constraint that the deforming inner surface flowed normal to itself at each sampled point in the flow. Vertex-wise thickness was estimated as the distance each inner surface vertex traveled over the course of the diffeomorphic flow to the outer surface, as shown in the bottom left panel of Fig. 1. Composite ERC and TEC thickness measures for each subject at each time point were estimated as the average vertex-wise thickness from the set of vertices in the inner surface correspondingly labeled as ERC or TEC respectively.

Rates of change in the ERC and TEC were computed as change in thickness per time (mm/year). For each subject, average atrophy rate was estimated by least squares fitting to the subject’s entire time series of smoothed measurements. As with amygdalar atrophy rates, ERC plus TEC rates were computed as a percentage change in thickness per year from baseline measurement. The combined ERC and TEC hypertemplate used in shooting was generated based on population surface generation (Ma et al., 2008).

### 2.3 Fixed effects modeling

Linear mixed effects (LME) and linear fixed effects (LFE) models (Bernal-Rusiel et al., 2013, Miller et al., 2013, Younes et al., 2014, Younes et al., 2014, Kulason et al., 2019, Kulason et al., 2020) have been used to represent the spatially normalized deformation marker, $J_{v}(\cdot )$, for a set of discrete points on anatomical surfaces for various groups of subjects. For each subject *s* in a cohort, $J_{v}(s)$ captures the local expansion/atrophy for subject *s* at point *v*. P-values are computed at the set of points, *V*, corrected for multiple comparisons (Nichols and Hayasaka, 2003) with covariates such as age, sex, and log intracranial volumes (icv). In the setting of two clinical groups (e.g. CU→CU and CU→MCI), the LFE models, under the null and alternative hypotheses, on $J_{v}(\cdot )$ at point *v* take the form

$$\begin{array}{c} H_{v}^{0}:\;J_{v}(s)=\alpha_{v}+\gamma_{v}d(s)+\delta_{v}i(s)+\kappa_{v}a(s)+{∊}_{v}(s)\\ H_{v}^{1}:\;J_{v}(s)=\alpha_{v}+\beta_{v}1_{\mathrm{MCI}}(s)+\gamma_{v}d(s)+\delta_{v}i(s)+\kappa_{v}a(s)+{∊}_{v}(s)\; \end{array}$$

respectively, for $1_{\mathrm{MCI}}(s)=1$ if s∈CU→MCI and 0 otherwise, and covariates d(s)=sex,i(s)=log-icv, and a(s)=age. The ${∊}_{v}(s)$ is the error term, assumed to be independent identically distributed Gaussian noise. The p-values are computed using random permutation (Nichols and Hayasaka, 2003) of the residuals, examining the global test statistic between the models under the null and the alternative hypotheses. This statistic is defined as

$$S^{*}=\underset{v}{\max}\left(S_{v}≔\ell_{v}^{H_{1}}-\ell_{v}^{H_{0}}\right),$$

where $\ell_{v}$ denotes the Gaussian log likelihood under each model. P-values are computed as the fraction of times $S^{*}$ is larger than the p-values obtained with the $S_{v}$’s calculated in the model at the maximum-likelihood parameter estimates of α and β. For this, we permute the residuals ${∊}_{v}^{0}(s)$ for the $H_{v}^{0}$ model with π a random permutation of the subjects, giving

$$J_{v}^{\pi }(s)=\alpha_{v}^{0}+\gamma_{v}^{0}d(s)+\delta_{v}^{0}i(s)+\kappa_{v}^{0}a(s)+{∊}_{v}^{0}(\pi_{s})\text{.}$$

Permutation testing delivers the familywise error rate (FWER), which we control for to deduce the subset of points for which the null hypothesis is rejected at 5% defined as $D=\{v:S_{v}⩾q*\}$ where q* is the 95th percentile of the observed value over the permutations (Nichols and Hayasaka, 2003).

Here, we use our estimated mappings from longitudinal diffeomorphometry (see Section 2.2.1) to compute for each subject *s* and the center of each triangle *v* on our hypertemplate surface, the deformation marker $J_{v}(s)$ as the log determinant Jacobian of the estimated mapping φ from initial to final time point in each subject’s time series. We compute the average and standard deviation of these markers across subjects within each group to compare relative atrophy between groups at each point on the amygdalar surface. We use fixed effects models with permutation testing to assess where in the amygdala this atrophy differs significantly between both CU→MCI and MCI→DAT groups versus the CU→CU group and compare the location of these regions to the spatial distribution of postmortem pathology.

### 2.4 Ex vivo tau reconstructions

#### 2.4.1 3D reconstruction of 2D digital pathology

Sets of 2D digital histology images at 2 μm resolution were mapped to the space of 3D high field ex vivo MRI at 0.125 mm resolution using Projective LDDMM (Stouffer et al., 2022). Each histology section is modeled as a Gaussian random field in the random orbit model (Grenander and Miller, 1998) with mean field modeled as an image in the orbit of a template $I_{\mathrm{temp}}$ under the group of diffeomorphisms, $I\in I=\{I=\varphi \cdot I_{\mathrm{temp}},\varphi \in G_{\mathrm{diff}}\}$. To accommodate differences in geometric dimension of digital histology images (2D) and MRI (3D), the mean field is given by the projection operation $P_{n}:I\;\mapsto \;J_{n}=P_{n}I+\mathrm{noise},n=1,\ldots ,N$, with observed target images $J_{n}$ modeled as conditional Gaussian random fields with mean $P_{n}I$. The projections $P_{n}I(y)$ are intrinsic to the histology process prescribing the weight an image value I(x) at location *x* contributes to the target image value J(y) at *y*. For serial sectioning at 1 mm intervals along the anterior-posterior axis of the brain:

$$\begin{array}{c} P_{n}I(y)=\int p_{n}(y,\mathrm{dx})I(x),\;y\in Y\subset R^{2}\\ \mathrm{with}\;p_{n}(y,\mathrm{dx})=\delta_{y,z_{n}}(\mathrm{dx}), \end{array}$$

where the Dirac delta measure, $\delta_{y,z_{n}}(\mathrm{dx})$, assigns nonzero measure only to the image value I(x) for $x=(y^{\left(1\right)},y^{\left(2\right)},z_{n})$, effectively restricting the template image to that within the image plane of target $J_{n}$ at location $z_{n}$ along the anterior-posterior axis of the brain. The tissue sectioning in histology introduces additional deformation modeled as 2D transformations $\phi_{n}$ in the 2D image plane, independent from section to section n=1,…,N:

(4) $$J_{n}=\phi_{n}\cdot P_{n}I+\mathrm{noise},n=1,\ldots ,N,$$

$$\mathrm{with}\;I=\varphi \cdot I_{\mathrm{temp}}\text{.}$$

Here, each $\phi_{n}$, is modeled as a diffeomorphism. We estimate φ and each $\phi_{n}$ as the flow of time-varying velocity fields, following the approach of LDDMM of Beg et al. (2005). We alternately optimize our cost function (5) for φ and each of the $\phi_{n}$ while holding the other fixed, as described in Stouffer et al. (2022). Estimation of φ is explicitly given for optimizing over $v_{t},t\in [0,1]$ to:

(5) $$\underset{{\left(v_{t}\right)}_{0⩽t⩽1}\in L^{2}([0,1],V)}{\inf}\int_{0}^{1}\|v_{t}{\|}_{V}^{2}\mathrm{dt}+\sum_{n=1}^{N}\|J_{n}-\phi_{n}\cdot P_{n}I{\|}_{2}^{2}$$

with $\|\cdot {\|}_{V}$ a norm defined over a space *V* of smooth time varying velocity fields and $\|\cdot {\|}_{2}$ the integral square norm. Estimation of each $\phi_{n}$ proceeds via estimation of velocity field ${\left(u_{t}\right)}_{0⩽t⩽1}\in L^{2}([0,1],V)$ with ${\dot{\phi }}_{t,n}=u_{t}\circ \phi_{t_{,}n},\;\phi_{0,n}=\mathrm{Id}$.

To resolve differences in the multi-scale resolution and contrast of the digital histology micro-scale images in relation to the tissue scale MRI, we expand the histology images using a Scattering Transform (Mallat, 2012). The Scattering Transform is comprised of an alternating sequence of convolutions with wavelets and non-linear modulus operations (i.e. modulus) that generate a sequence of multi-scale images $S:J\;\mapsto \;(S_{J}^{1},\ldots ,S_{J}^{48})$, which reflect the tissue characteristics originally encoded at the high resolutions of digital pathology. We use Principal Component Analysis (PCA) to build a 7-dimensional linear predictor from the scattering images with a constant image (Stouffer et al., 2022), which approximates an MR contrast image of histology as a function of the predictor $J_{n}(\alpha ),\alpha =\alpha_{1},\ldots ,\alpha_{7}$. The optimization problem simultaneously solves for the diffeomorphism transforming the template onto the target histology with (5) and the low-dimensional linear predictor, with estimation of $\alpha_{n}$ following least squares minimization: $\min_{\alpha \in R^{7}}\|J_{n}(\alpha )-\phi_{n}\cdot P_{n}I{\|}_{2}^{2}$ (Stouffer et al., 2022).

For distortion extending to tears, tissue folding, and other artifacts, we introduce a set of latent variables in the context of Gaussian mixture models, assigning each pixel location in the image plane to one of three classes: foreground tissue, background tissue, or artifact, as described in Tward et al. (2020). Estimation of latent variable values together with each $\phi_{n}$ is achieved with the Expectation–Maximization (EM) algorithm (Dempster et al., 1977), resulting in a cost function with matching term weighting each location in the image plane according to its iteratively estimated posterior probability of being foreground tissue (Stouffer et al., 2022).

The high field MRI was collected in three blocks. To integrate measures across blocks, we used an in-house tool to rigidly align each set of three MRI blocks. The Mai Paxinos Atlas (Mai et al., 2008), was rigidly aligned to a surface rendering of the complete hippocampal geometry of each brain sample manually to a surface rendering of the Mai hippocampus. The findings reported in Section 3 reflect the coordinate system of the Mai atlas.

#### 2.4.2 Neurofibrillary tau tangle density measures

We quantified tau pathology from digital histology images as number of neurofibrillary tau tangles (NFTs) per cross-sectional tissue area. Measures of NFT density were computed over each section following the approach described previously (Stouffer et al., 2022). For each brain sample, a UNET (Ronneberger et al., 2015) with architecture specified in Supplementary Section Appendix B, was trained on a subset of patches of background and foreground tissue with NFTs manually annotated. Separate UNETs were trained for each brain sample to account for differences in staining intensity that resulted from executing the same protocol at different times by different handlers. Resulting probability maps predicted the likelihood of each pixel’s being part of an NFT. Probability maps were then segmented using an available implementation of the Watershed algorithm (Bradski, 2000) to delineate individual NFTs. Each histology image was segmented into foreground tissue and background using Otsu’s method (Otsu, 1979), and final measures of NFT density per slice, region, or subregion were computed by taking a sum of NFTs within foreground tissue divided by the square mm area of foreground tissue in the region of interest.

#### 2.4.3 3D reconstruction of NFT densities

Distributions of tau pathology in 3D were reconstructed per brain sample following estimation of geometric mappings between histological images and 3D MRI (see Section 2.4.1). Estimated NFT densities within each image plane were modeled as features, $f_{i}$, associated to “particles” at locations, $x_{i}$, in a regular voxel grid, using a measure-based framework, as described in Miller et al. (2021). Weights, $w_{i}$, reflecting the square mm area of tissue captured by the *i*th particle at each position $x_{i}$ were associated with the particle measures (mathematical measures, see Miller et al. (2021)) and carried to the Mai coordinate space, together with MRI and associated MTL segmentations, following the prescribed action of diffeomorphisms on measures (Miller et al., 2021).

In the Mai coordinate space, measures were pooled across histological sections and resampled within the dense volume. Physical and feature space were transformed independently, as enabled by the decomposition of measures into a physical density (cross-sectional tissue area (mm^2^) per unit of 3D space) and conditional feature probability (number of 2D detected NFTs in unit of 3D space given its physical density). Spatial resampling to a regular grid within the volume of the Mai atlas at a resolution of MRI was achieved with a Gaussian kernel, assigning a fraction of each particle measure’s weight, $w_{i}$, to each new particle x′ with new weight, $w_{i}^{\prime }$ in the resampled space. Feature values for each new particle x′ were computed as the expected first moments according to the empirical distribution defined by the spatial reassignment of weights.

#### 2.4.4 Laplace–Beltrami resampling to surfaces

In addition to resampling within the dense volume, resampling along the surface manifold of MTL subregions was achieved with a nearest neighbor kernel, “projecting” NFT density measures to the boundary of each surface by assigning the entirety of each particle’s tissue area within a given region at high resolution to a single particle within the manifold. Smoothed NFT densities were estimated as the ratio of smoothed NFT counts (gτ^) to smoothed cross-sectional tissue areas (ga^) following expansion of each vertex defined function $g_{\tau }(x_{i})≔f_{i},g_{a}(x_{i})≔w_{i}$ in the Laplace–Beltrami basis, as described previously (Qiu et al., 2006, Stouffer et al., 2022). Specifically, for each of $g=g_{a}(\cdot )$ and $g=g_{\tau }(\cdot )$, we compute its smoothed approximation, g^=g^a(·),g^=g^τ(·), as:

argming^‖g^-g‖22+k‖∇g^‖22=∑i=1N〈g,βi〉Vβi(·)1-kλiw(·),with〈g,βi〉V≔∑y∈Vβi∗(y)g(y)w(y)

where $B≔\{\beta_{1},\cdots ,\beta_{N}\}$ is a basis for the Laplace–Beltrami operator, w(·) defines a weight for each vertex based on partitioning the surface area of adjoining faces, and *k* is the smoothing constant, with value of 2 in the results presented in Section 3.

## 3 Results

### 3.1 Spatial distribution of amygdalar atrophy

As described in Sections 2.2.1, 2.3, we estimated mappings of a common hypertemplate of the amygdala and of the combined ERC + TEC to each subject’s trajectory and the consequent flow from start to end scan time through this trajectory. Using this method, we have previously reported the spatial distribution of thickness change observed in the ERC + TEC in subsets of both the ADNI and BIOCARD datasets, with greatest shape change observed anteriorly in the ERC + TEC (Kulason et al., 2019, Kulason et al., 2020). Here, we compare the magnitude and the spatial distribution of atrophy within the amygdala across our three groups.

**Fig. 3:**
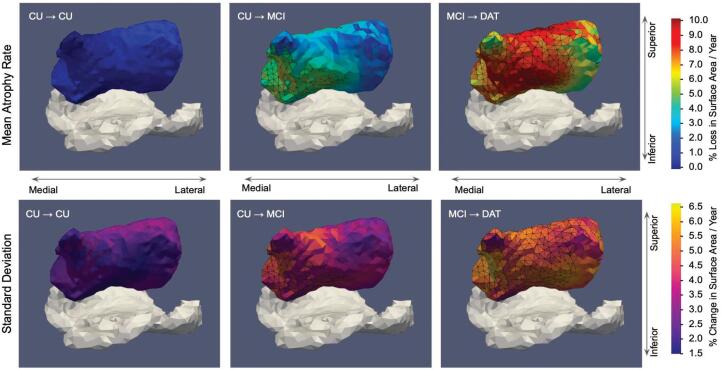
Mean per triangle percent atrophy rate in the amygdala in CU→CU, CU→MCI, and MCI→DAT cohorts (n=33,n=16,n=18 respectively) (top). Standard deviation of per triangle percent atrophy rate in the amygdala shown in bottom row. Percent atrophy rate computed as percent change in surface area per year $\left(100\cdot \frac{A_{\mathrm{final}}-A_{\mathrm{initial}}}{A_{\mathrm{initial}}\cdot (t_{\mathrm{final}}-t_{\mathrm{initial}})}\right)$, estimated following longitudinal diffeomorphometry. Average percent atrophy rate over entire surface for CU→CU, CU→MCI, and MCI→DAT groups are 0.94,3.58 and 7.51%, respectively. Average per triangle standard deviation over entire surface for three groups are 2.53,3.7, and 4.79%, respectively. Triangles exhibiting atrophy rates significantly different from those in the CU→CU group after controlling for FWER are outlined in black (0/1696, 526/1696, and 1345/1696 in three groups, respectively). ERC shown in grey for reference.

For comparison to atrophy rates per individual subject, described in the following section, we estimated the atrophy per year on each triangle of the template amygdala for each subject’s trajectory as the determinant Jacobian of this subject-specific flow from first to last scan time divided by the difference in these times. We consequently computed the average atrophy rate at each triangle on the amygdala surface for each group of subjects. Fig. 3 shows the average atrophy rate at each triangle on the hypertemplate amygdalar surface for CU→CU, CU→MCI, and MCI→DAT groups and corresponding standard deviation of this atrophy rate at each triangle within each group. Average atrophy rate ± standard deviation across triangle regions within the CU→CU, CU→MCI, and MCI→DAT groups are 0.94±2.53%,3.58±3.7%, and 7.51±4.79%, respectively.

As described in Section 2.3, we used fixed effects modeling with permutation testing to control for the FWER and select the set of triangles for which the model under the null hypothesis is rejected with a p-value of 0.05. This yielded an assessment of *where* within the amygdala the reported average atrophy rate of the CU→MCI and MCI→DAT groups (Fig. 3) was significantly different than that of the CU group, which we used to compare these in vivo results to ex vivo tau reconstructions (see Section 3.4).

### 3.2 Amygdalar and entorhinal individual atrophy rates

**Fig. 4:**
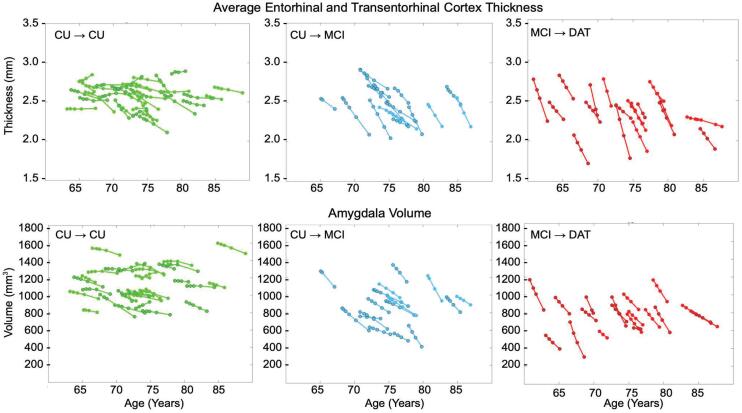
Smoothed measures of ERC plus TEC thickness (top) and amygdala volume (bottom) plotted against subjects’ ages at each scan time point. CU→CU group in green (left), CU→MCI in blue (middle), and MCI→DAT in red (right). Female patients outlined with black circles.

Following estimation of a smoothed trajectory over time for each subject via longitudinal diffeomorphometry (see Section 2.2.1), this trajectory was resampled at each subject’s each original scan time point, enabling the assessment of each individual’s atrophy over time. The results of this resampling are plotted for each subject in Fig. 4. Average atrophy rates for ERC thickness and amygdala volume were computed from the smoothed measures for each series of time points for each subject via least squares fitting. Scan periods ranged from 1 year to 5.1 years for sets of time points, and each subject had between 3 and 7 scans. In both entorhinal and amygdalar regions, those in the CU→MCI and MCI→DAT groups exhibited higher atrophy rates than stable controls. Mean atrophy rates of the ERC plus TEC were 1.1,3.8, and 7.0% thickness loss per year, and of the amygdala, were 1.5,6.8, and 11.6% volume loss per year for CU→CU, CU→MCI, and MCI→DAT subjects, respectively. These are recorded with standard deviations in Table 3.

**Table 3:** Mean and standard deviation of percent atrophy rate in the combined ERC and TEC and in the amygdala for each of three groups. Percent atrophy rate computed as percent change in thickness or volume per year, respectively, for ERC + TEC and amygdala.

	**CU**→**CU**	**CU**→**MCI**	**MCI**→**DAT**
Mean Entorhinal Atrophy Rate	1.1±1.6%	3.8±1.9%	7.0±3.4%
Mean Amygdalar Atrophy Rate	1.5±2.0%	6.8±4.1%	11.6±5.8%

**Fig. 5:**
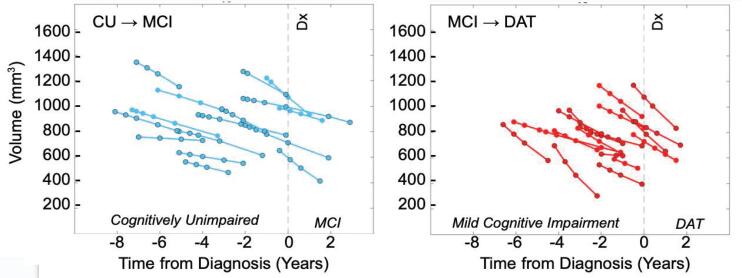
Smoothed measures of amygdala volume plotted against relative time to change in cognitive status, established by a diagnosis of MCI or AD dementia, respectively, in each of the CU→MCI and MCI→DAT groups.

To assess the potential of amygdalar atrophy rate, measured via 3T MRI, as a biomarker for AD, we illustrate the estimated volume trajectories following smoothing in comparison to the subjects’ change in cognitive status. Fig. 5 shows these trajectories for both CU→MCI and MCI→DAT groups, with volume loss observed as early as 7–8 or 6–7 years prior to diagnosis in each group, respectively. We additionally compared distributions of estimated atrophy rates in the CU→CU group to those of the CU→MCI and MCI→DAT groups via receiver operating curve (ROC) analysis to estimate a distinguishing cutoff between groups. This yielded an optimal threshold of discrimination between CU→CU subjects and both CU→MCI and MCI→DAT subjects at 4.7% volume loss per year, with sensitivity of 0.91, specificity of 0.91, and AUC of 0.96.

For comparison to other studies, we also analyzed smoothed trajectories of ERC + TEC thickness and amygdala volume in a subset of the CU→MCI cohort with confirmed Aβ positive status at baseline. Mean atrophy rates for the ERC + TEC and amygdala in this subset were estimated as 2.52±0.98% and 5.76±1.81%, respectively. See Supplementary Section Appendix C for details.

### 3.3 Geometric reconstructions of postmortem brain samples

**Fig. 6:**
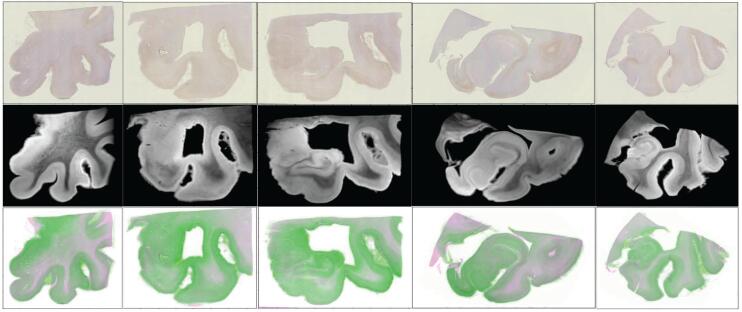
Visual comparison of 2D histology slices (top row) and corresponding estimated 2D slices of deformed 3D MRI to histology coordinates (middle row). Select slices taken from the first two blocks of tissue and ordered left to right in rostral to caudal order. Overlap between estimated deformed MRI slice (green) and PHF-1 image (magenta) illustrates areas of match and mismatch between tissue boundaries (bottom row).

**Fig. 7:**
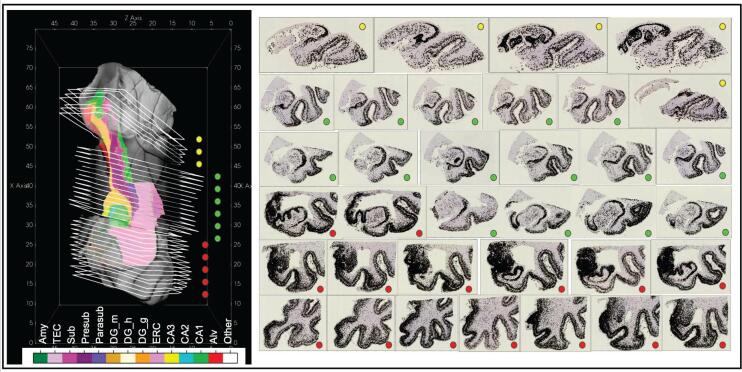
Complete sets of digitized PHF-1 stained histology sections for 3 blocks of MRI for an AD brain sample. 3D MRI shown with manual segmentations of MTL subregions (left). Boundary of each histological section on right shown in white in position following transformation to 3D space (left). Detected NFTs plotted as black dots over each histology slice (right).

**Fig. 8:**
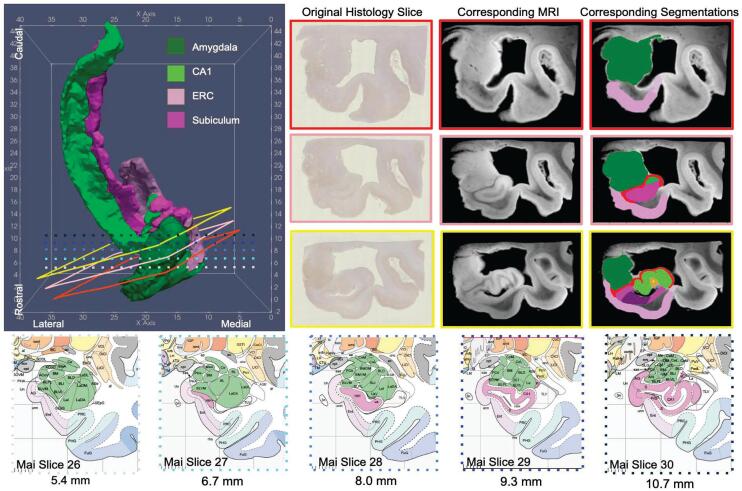
3D Reconstruction (left) of 4 MTL subregions for an AD brain sample in the coordinate space of the Mai Paxinos Atlas. Example section of histology and corresponding MRI slice (right) shown at approximate location of intersecting coronal planes taken from the pages of the Mai Atlas (bottom).

Two sets of 35 digitized histological sections stained with PHF-1 were aligned to 3D high field MRI blocks of corresponding MTL tissue following the approach described in Section 2.4.1. Alignment accuracy was evaluated qualitatively by visual comparison of overlap between deformed MRI and histological images (see Fig. 6, and quantitatively by comparing 2D segmentations of MTL subregions on histological images to 3D segmentations of corresponding regions deformed to 2D. Dice Score and 95th Percentile Hausdorff distance for each region on each slice of one brain were computed with average overlap scores of 0.85, 0.82 and average 95th percentile Hausdorff distance of 1.886 mm and 1.039 mm for the amygdala and ERC, respectively. Positions of each section within each block are shown for one brain sample in Fig. 7 with manual segmentations of MTL subregions illustrated across the rigidly aligned MRI blocks. Following alignment of 2D histological sections to 3D MRI, both sets of images were transported to the space of the Mai Paxinos atlas (Mai et al., 2008) with estimation of rigid alignment between hippocampal surfaces for each brain sample and that of the atlas. Fig. 8 exhibits surface renderings of select MTL subregions for one brain sample in the coordinate system of the Mai Paxinos atlas. Snapshots of the atlas show coronal slices in the area of the anterior MTL intersecting with select histological slices.

### 3.4 NFT densities in advanced AD

**Fig. 9:**
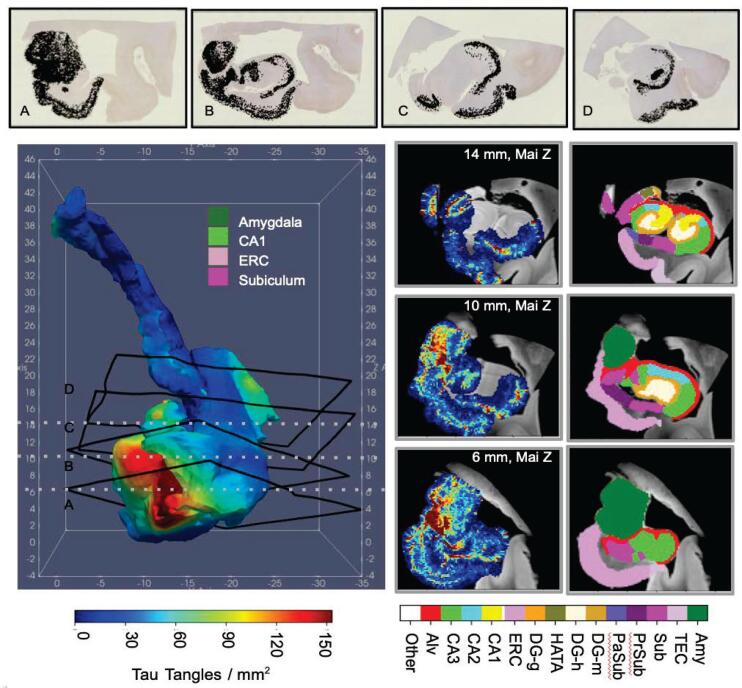
Distributions of NFT densities computed from digital histology at 2 μm (top) reconstructed in 3D for an advanced case of AD. Densities within a subset of regions of MTL (amygdala, ERC, CA1, and subiculum) sampled within the dense metric of the 3D MRI at 0.2 mm resolution (right) or projected to the surface of each structure and smoothed with the Laplace–Beltrami operator (left). Sections of MRI shown at 6, 10, and 14 mm, corresponding to coronal slices in the Mai Paxinos atlas.

**Fig. 10:**
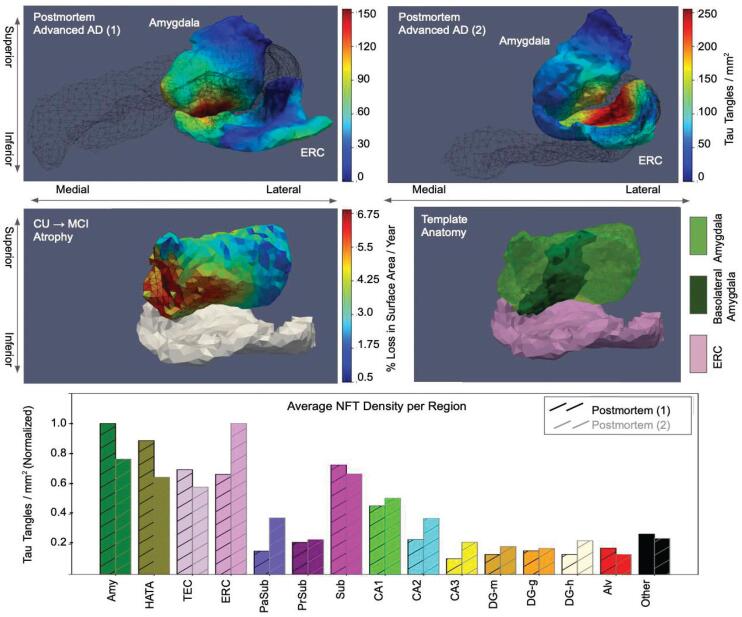
Posterior view of amygdala-ERC boundary in hypertemplate (middle) and two advanced AD postmortem samples (top). NFT densities for postmortem samples shown following projection to each surface and smoothing with the Laplace–Beltrami operator. Outline of CA1 and subiculum surfaces for postmortem samples shown in black mesh (top). Mean per triangle percent atrophy rate in the amygdala for CU→MCI group (middle left). Edges outlined in black for triangles with average amygdala atrophy rate that differ significantly from those of CU→CU group after controlling for FWER at 5%. Basolateral amygdalar subregion delineation shown in hyptermplate (middle right). Average NFT density within each MTL subregion normalized to maximum average for each AD brain sample (bottom).

NFTs were detected on each histological image for each brain sample with separate UNETs trained independently on a subset of training data for each sample. We evaluated the accuracy of UNET-predicted per-pixel probability of tau using 10-fold cross validation. For the two brain samples, mean area under curve (AUC) was estimated at 0.9860 and 0.9873, and mean accuracy at 0.9729 and 0.9546, respectively (see Supplementary Section Appendix B for details). We evaluated the accuracy of discrete NFT detections following segmentation with the watershed algorithm by direct comparison of the number of NFT detections to the number of NFTs manually annotated in a set of ten image patches reserved for validation. The number of NFT detections varied on average 19±1% from the number of annotated NFTs across the set of patches, with similar trends in both numbers of NFTs from patch-to-patch, ordered anterior to posterior, exhibiting similar relative measures in density between predictions and annotations. Further details and exact measures provided in Supplementary Section Appendix B. 2D measures of NFT density were carried to the space of the Mai Paxinos atlas, together with MRI and manual segmentations of the MTL. NFT density measures were pooled across sections and resampled at 0.2 mm resolution within the coordinate space of the Mai atlas (see Fig. 9). Deformation of 3D MTL segmentations to 2D histology images enabled assignment of NFTs into MTL subregion. NFT densities per MTL subregion were computed overall (see Fig. 10) and over the surface boundary of each region (see Fig. 9), following smoothing with the Laplace–Beltrami operator, as described in Section 2.4.4.

NFT densities are reported on different scales for each brain sample. To compare NFT densities across brain samples, subsets of 4–5 additional sections from each brain were selected in the rostral hippocampus at approximate locations of original sections. These two subsets were stained simultaneously to achieve consistency between them and the respective UNET trained on the original training data from each brain was used to detect NFTs in these sets of replicate slices. Ratios of NFTs detected on the original and new version of each section were computed, yielding average ratios of 2.7 and 27.2 for brain samples from subjects 1 and 2, respectively. These differences in level of detection speak to the effect that variation in staining intensity, timing, and handling of tissue samples has on absolute counts of NFTs. Therefore, to compare relative distributions of tau tangles between the two brain samples (see Fig. 10), we normalized NFT density measures accordingly in each brain to the range 0 to 1. Average NFT densities per MTL subregion showed highest amounts of NFTs in amygdala, hippocampal-amygdalar transition area (HATA), ERC, CA1, and subiculum for both advanced AD samples (see Fig. 9, Fig. 10). Indeed, highest NFT densities within the ERC, amygdala, and HATA constitute a propensity of NFTs within the rostral third of the MTL.

Tau pathology localized not just *to* particular regions (e.g. amygdala, ERC, CA1, subiculum), but also within them. As illustrated in Fig. 10, high densities of NFTs in amygdala and ERC concentrated particularly at the border between the two structures. Within the amygdala, tau was concentrated at the inferior, medial boundary of the amygdala, particularly in its basomedial and basolateral regions, with the lateral region largely devoid of pathology. These high pathology regions were also those regions showing significant atrophy in both CU→MCI and MCI→DAT diagnostic groups after permutation testing and control of FWER at 5% (see Section 2.3). As illustrated in Fig. 3, Fig. 10, approximately $\frac{1}{3}$ of the amygdala was found to have atrophy significantly different in the CU →MCI group than in the CU→CU group (526/1696 triangles), with all of these regions concentrating to the medial, and inferior-medial regions of the amygdala. Within the DAT group, a large majority of the amygdala (1345/1696 triangles) exhibited atrophy significantly different from that of the CU→CU group, with the lateral region, as in observed distributions of ex vivo pathology, being the region without such atrophy (see Fig. 3).

## 4 Discussion

There are two primary findings from this research. First, these results demonstrate that the amygdala atrophies at a significantly faster rate in subjects progressing from CU to MCI and from MCI to AD dementia, compared with those who remain CU. In both the ERC and amygdala, our examined CU→CU group exhibited some level of atrophy (1.1% thickness loss and 1.5% volume loss, respectively). Likely an aspect of normal aging independent of disease, these atrophy rates nevertheless suggest that these subjects, if they live long enough, might develop cognitive impairment given the accumulated atrophy of the ERC and amygdala. However, the differences between those in CU→MCI and CU→CU in volumetric atrophy rate as measured by MRI from a series of 3–6 scans are comparable to those in thickness atrophy rate of the ERC plus TEC, with CU→MCI individuals atrophying at a rate over 3x that of CU→CU individuals, and MCI→DAT individuals at a rate over 6x that of CU→CU individuals. In terms of onset, we have previously shown that the MTL as a grouped structure including ERC, TEC, and amygdala exhibits a changepoint nearly a decade before clinical time (Younes et al., 2019). The findings here explicitly analyzing amygdala and ERC separately in individuals converting from CU to MCI and from MCI to AD dementia indicate an early role for this MTL aggregate marker.

Second, these results demonstrate concordance between patterns of microscopic NFT pathology, specific to pathologically confirmed AD, and macroscopic ex vivo measures of atrophy through reconstruction of microscopic measures in the dense, submillimeter metric of high field MRI. Previous studies have shown correlation between single time point ex vivo measures of thickness and levels of NFT and TDP-43 pathology, particularly in the areas of ERC and hippocampus (Ravikumar et al., 2021, Llamas-Rodríguez et al., 2022). Here, we show significant atrophy rates measured longitudinally in vivo in both the ERC and amygdala, which are correspondingly supported by our observation of highest densities of NFTs in our postmortem specimens not only in the ERC, but also in the amygdala. Furthermore, the variation in level of atrophy seen over the surface of the amygdala (Fig. 3) and previously reported by our group and others in the ERC (Kulason et al., 2020, Holbrook et al., 2020) shows highest and significantly different atrophy in the medial amygdala and anterior ERC, in CU→MCI and MCI→DAT subjects compared with CU→CU individuals, which echo the areas where we observed highest NFT densities postmortem (Fig. 10). These findings are consistent with the ex vivo correlation observed in the anterior ERC in Ravikumar et al. (2021) and with those in the ERC and amygdala observed in Yushkevich et al. (2021). Together, our coupled in vivo and ex vivo results suggest that such NFT pathology is underlying observed atrophy. Also, as MTLs from advanced cases of AD, these reconstructed distributions exhibiting highest end stage densities in ERC, HATA, and amygdala (see Fig. 10) suggest that these regions are involved throughout the course of disease to accumulate such high end stage densities. The spatial organization of these structures further suggests that one may consider AD as a preferentially rostral disease within the MTL. However, further studies across the full spectrum of AD progression will be needed to establish the exact timing and progression of this spatial segregation.

The emergent role of the amygdala, here, is supported by our own work in shape diffeomorphometry (Miller et al., 2015a, Miller et al., 2015b) as well as recent studies in MRI (Wang et al., 2021), tau PET (Berron et al., 2021), and tau pathology (Yushkevich et al., 2021) that have seen similarly high levels of atrophy or tau pathology in the amygdala, particularly in early AD. Further work is needed to sufficiently delineate which areas of the amygdala might be more involved in early AD, but in both brain samples analyzed here, basolateral and basomedial regions of the amygdala, often classified together with the lateral nucleus as “core” amygdala (Sheline et al., 1998), demonstrated higher tau densities as those adjacent to the ERC (Fig. 10). These regional findings echo those found both in postmortem histological analysis (Arnold et al., 1991) and more recently, the 3D reconstruction of postmortem pathology in Yushkevich et al. (2021), which both cite the accessory basal nucleus, in particular, as regions of high NFT pathology. In addition, as highlighted above, both CU→MCI and MCI→DAT groups showed on average higher levels of atrophy, differing significantly from that in controls, within the medial inferior region of the amygdala in contrast to the lateral superior region (see Fig. 3). Evidence of differential involvement of amygdalar nuclei has been cited in other neuropsychiatric diseases, such as Parkinson’s Disease (Harding et al., 2002) and depression (Sheline et al., 1998). Following Amunts et al. (2006), we are currently segmenting subregions of the amygdala in high field MRIs of MTLs from subjects with early and advanced AD to further assess specificity of tau pathology for particular amygdalar nuclei in the context of AD.

This work has both strengths and limitations. It is foremost one of few studies integrating microscopic *and* macroscopic scales of both spatial *and* temporal analysis through digital pathology and longitudinal MR imaging measures. Having developed the technology and mathematical infrastructure to integrate these two data types, we are poised to analyze other cohorts to corroborate the putative link between amygdala and ERC atrophy observed in MR imaging to underlying NFT pathology.

Regarding in vivo analysis, many previous clinical imaging studies from our own group as well as others (Miller et al., 2015a, Miller et al., 2015b, Wang et al., 2021, Berron et al., 2021) have compared control and disease groups only cross-sectionally at a single time point. The longitudinal analysis here tracks subject specific measures over time. As such, it reduces variance inherent to the use of single measures, such as single time point thickness, to indicate disease status. Similar to the findings presented here in the ERC, others have reported significant differences in atrophy rate, particularly of the anterior-lateral entorhinal region, across three similar groups of the ADNI dataset (Holbrook et al., 2020). Further longitudinal analysis amongst additional datasets will provide greater insight into the exact timing, spatial distribution, and progression of both ERC and particularly amygdala involvement in AD, as necessary for the development of biomarkers and understanding the emerging role of the amygdala in AD’s early stages.

Regarding ex vivo analysis, other efforts to reconstruct distributions of tau pathology in 3D include efforts to analyze concordance of distributions between subjects in early AD (Ravikumar et al., 2021, Yushkevich et al., 2021, Llamas-Rodríguez et al., 2022) and to develop validation measures for in vivo molecular imaging (Ushizima et al., 2022). These studies harbor advantages in terms of number of samples (Ravikumar et al., 2021, Yushkevich et al., 2021, Llamas-Rodríguez et al., 2022) and scope of brain region (e.g. whole brain) (Ushizima et al., 2022) analyzed. Here, we have focused our methods and analysis on reconstructing the 3D distribution of tau pathology within single subjects in a coordinate system (e.g. over the surface boundary of MTL substructures) consistent with identifying corresponding shape changes as measured in clinical subjects. Hence, we differ from some of these previous studies in providing a quantitative metric of pathology (NFT density as number of NFTs per square mm of tissue) over the unique surface geometry of each subject’s individual MTL structures rather than average measures in a common atlas. Furthermore, through mapping to 11T MRI, we achieve sub-millimeter resolution of pathology measures, maintaining much of the precision found at microscopic scales and far exceeding the resolution of contemporary PET imaging (Ushizima et al., 2022). In addition, we have measured tau pathology with the PHF-1 antibody whereas most previous studies have used AT8, thus leading to slightly different staining patterns and consequently, levels and types of tau pathology presumably detected and reconstructed.

Finally, manual segmentations of regions of the MTL in both in vivo MR images and ex vivo MRI offer an improvement over past efforts analyzing amygdalar atrophy and pathology in which segmentations were estimated from alternative templates (Kulason et al., 2019, Yushkevich et al., 2021, Fischl et al., 2002). While automatic segmentation protocols of amygdalar or entorhinal atrophy rate, as measured by MRI, would be optimal for measuring large numbers of individuals, existing algorithms have not yet achieved accuracy near that of manual segmentation. Indeed, the amygdala has been cited as a structure harder to segment than others in the MTL (Jack et al., 1997), and recent comparisons of Freesurfer, FIRST, and ANTS segmentations of the amygdala to manual segmentations achieve Dice overlap scores only between 0.6 and 0.7 (Alexander et al., 2020). A potential avenue for future work is the use of our manual segmentations to develop an accurate automatic segmentation algorithm. Yet, the development of more accurate automatic segmentation schemes is an active area of research, for which we hope the marked differences in atrophy rate presented here will continue to motivate the field to achieve, in particular, more consistent segmentations of the amygdala, ERC, and TEC.

Limitations of this study include both lack of data points (e.g. number of ex vivo samples) and restricted geometric diversity within in vivo cohorts. Here, we present ex vivo 3D reconstructions of MTL NFT pathology from only two brain samples. Other studies have reconstructed distributions of pathology amidst larger cohorts, which has enabled the use of statistical methods to extract patterns in distributions across subjects (Yushkevich et al., 2021) and correlate these patterns with other measures such as cortical thickness (Ravikumar et al., 2021, Llamas-Rodríguez et al., 2022). However, these cohorts have increased the heterogeneity in diagnosis and both stage and type of pathology observed. In contrast, we present results of two cases both with neuropathological diagnoses of AD and equivalent staging of tau and Aβ pathology. Nevertheless, the reconstructed distributions provide a snapshot only of the relative distribution of tau pathology at the end course of disease, which precludes direct comparison to the spatial atrophy measured in our in vivo cohorts across these different stages. However, the relative lack of pathology observed in certain regions (e.g. body of hippocampus, lateral and superior portions of the amygdala) even at this late stage of disease suggests these areas might remain largely devoid of pathology throughout the stages of disease. This hypothesis fits with the gradual spread and continued accumulation of pathology described by Braak and Braak (1991) as opposed to a marked appearance and then disappearance of pathology. Indeed, Yushkevich et al., analyzing a cohort comprised primarily of *early* AD and mixed types of pathology, not only report highest NFT burdens in the amygdala and ERC over other areas of the MTL, as found in our two cases (see Fig. 10), but also highlight differing levels of pathology throughout the hippocampus (Yushkevich et al., 2021). Specifically, they report highest NFT burdens in both CA1 and subiculum, as evidenced in our cases, and furthermore, demonstrate a similiar lack of pathology in what they segment as posterior hippocampus, with both anterior counterparts and the tail of the hippocampus manifesting higher levels of pathology, as in our reconstructions (see Fig. 9). To build further support for the specificity of clinical imaging biomarkers such as MRI-measured ERC and amygdala atrophy rate in signature patterns of pathology in AD, we are currently reconstructing the distributions of both NFT and Aβ pathology in early, intermediate, and advanced stages of AD, following the methods outlined here. Future work could include the use of different antibodies (e.g. CP13) (Koss et al., 2016) in settings of earlier stage individuals for better detecting apparent pathology at these stages (e.g. pre-tangles and NFTs) and reconstructing the relative distribution in these individuals in 3D space.

In our in vivo analysis, we restricted our attention to individuals with an anteriorly continuous CS to achieve accurate and consistent segmentation of entorhinal and transentorhinal regions across subjects. We are currently extending our segmentation protocol for delineation of anteriorly discontinuous CS in a manner consistent both with the unique geometry of these cases and our protocol for the continuous case so that in future studies, we will maximize our analysis by comparison across all types of geometries. Additionally, given the effort involved in manually segmenting each structure accurately (1-2 hours per structure per scan), atrophy rates were assessed for ERC and amygdala only in the left cerebral hemisphere. In contrast, availability of postmortem samples dictated NFT reconstructions to be computed for each of one left and one right hemisphere. Previous studies have suggested differences of involvement in right versus left amygdalas (Yue et al., 2016). Future work will be needed to confirm or deny any differences in pathology versus atrophy in each hemisphere, using the manual segmentation protocol described here to avoid known biases in automatic segmentation protocols between right and left sided amygdalas, in particular (Alexander et al., 2020).

Finally, this work has aimed to specifically link pathology at the micron scale to longitudinal MR imaging markers from clinically well-characterized groups. In addition to investigating 3D distributions of both NFT and amyloid pathology, future work could include the association of measured atrophy rate to CSF biomarker measures of amyloid and tau in subsets of individuals progressing from CU to MCI. This would enable further characterization of the specificity of these rates to “preclinical AD” and to amyloid and tau status. Additionally, the immense increase in both robustness and availability of diverse imaging technologies including diffusion tensor imaging and molecular imaging (e.g. Tau and Amyloid PET) (Leuzy et al., 2019, Villemagne et al., 2018) over the last decade has already begun to enable the collation of longitudinal datasets with more advanced and diverse types of images. Such datasets include OASIS-3 (LaMontagne et al., 2019) and the developing Alzheimer’s Disease Connectome Project (Van Essen and Glassers, 2016). Concordant longitudinal analysis, as presented here, of cohorts in these datasets and across different molecular imaging modalities in the future will offer the potential for corroborating and coordinating biomarkers across multiple modalities.

In conclusion, we have demonstrated the existence of significant atrophy in *both* the ERC and amygdala in early stages of MCI and Alzheimer’s dementia. Measurable from a series of MR scans and putatively linked to high levels of AD specific pathology, atrophy rates in both ERC and amygdala present as viable biomarkers that may someday be used for both early diagnosis and management of AD.

## Funding

This work was supported by the National Institutes of Health (U19-AG033655, P30-AG066507, P41-EB031771, R01-EB020062 (MM), T32-GM13677 (KS), U19-MH114821, R01-NS074980-10S1, RF1MH126732, RF1MH128875, RF1MH28888 (DT), the Kavli Neuroscience Discovery Institute (KS, DT, MM), and the Karen Toffler Charitable Trust (DT).

## CRediT authorship contribution statement

**Kaitlin M. Stouffer:** Conceptualization, Methodology, Software, Formal analysis, Investigation, Data curation, Writing – original draft, Writing – review & editing, Visualization. **Claire Chen:** Validation, Investigation, Data curation, Writing – review & editing. **Sue Kulason:** Formal analysis, Visualization, Writing – review & editing. **Eileen Xu:** Validation, Investigation, Data curation. **Menno P. Witter:** Methodology, Validation, Investigation, Writing – review & editing, Supervision. **Can Ceritoglu:** Software. **Marilyn S. Albert:** Writing – review & editing, Supervision, Funding acquisition. **Susumu Mori:** Resources. **Juan Troncoso:** Validation, Resources, Data curation, Writing – review & editing. **Daniel J. Tward:** Methodology, Software, Writing-review-editing. **Michael I. Miller:** Conceptualization, Methodology, Writing – original draft, Writing – review & editing, Supervision, Funding acquisition.

## Declaration of Competing Interest

The authors declare the following financial interests/personal relationships which may be considered as potential competing interests: MM and SM are co-owners of Anatomy Works and are entitled to royalty distributions from the company, with the arrangement being managed by Johns Hopkins University in accordance with its conflict of interest policies.
