# Supplementary material for: Early amygdala and ERC atrophy linked to 3D reconstruction of rostral neurofibrillary tau tangle pathology in Alzheimer’s disease
Source: Neuroimage Clin. 2023 Mar 15;38:103374. doi: 10.1016/j.nicl.2023.103374 (PMC10034129; doi:10.1016/j.nicl.2023.103374)
Supplement: Supplementary data 1 [file mmc1.pdf]

Appendix A. Variability Measures in Manual Segmentation

Variability in manual segmentations of MTL structures was assessed by computing Dice overlap scores in replicate segmentations of the same structure drawn by the same individual at different times. Table A.1 shows individual overlap measures computed for a subset of scans and subjects from each of the three cohorts (CU $\rightarrow$ CU, CU $\rightarrow$ MCI, MCI $\rightarrow$ DAT) analyzed in this work. Average overlap in any pair of segmentations of the same scan was 0.87 ± 0.04.

Subject and Scan	Overlap Trials 1 and 2	Overlap Trials 2 and 3	Overlap Trials 1 and 3
CU→CU-A T1	0.91	0.91	0.87
CU→CU-A T2	0.91	0.92	0.89
CU→CU-B T1	0.90	0.90	0.89
CU→CU-B T2	0.90	0.91	0.89
CU→MCI-A T1	0.88	0.87	0.91
CU→MCI-A T2	0.85	0.89	0.87
CU→MCI-B T1	0.92	0.86	0.87
CU→MCI-B T2	0.88	0.88	0.86
MCI→DAT-A T1	0.87	0.83	0.77
MCI→DAT-A T2	0.78	0.86	0.84
MCI→DAT-B T1	0.82	0.83	0.80
MCI→DAT-B T2	0.81	0.78	0.86
Average	0.87 ± 0.4	0.87 ± 0.4	0.86 ± 0.4

Table A.1: Overlap measures between replicate segmentations of same structure in same scan by same individual. Subjects denoted by cohort abbreviation and letter code (A,B). Scans denoted by time point (T1, T2). Trial 1 and trial 2 were separated by 4 days; trial 2 and trial 3 by 7 days.

Appendix B. UNET Details

Individual NFTs were detected from digital histology images of tissue samples stained with PHF-1. Detection followed a two-step algorithm: 1) prediction of each pixel's probability of being part of an NFT and 2) segmentation of probability maps into discrete connected components corresponding to NFTs. The architecture of the UNET trained to predict the probabilities in step 1 is shown in Table B.1.

Table B.1: Structure of UNET predicting per-pixel probabilities of inclusion within NFT. The left 3 columns describe the contraction layers, and the right 3 columns describe the expansion layers. The number of parameters correspond to those associated with linear filters + bias vector. Conv: 3×3 convolution with stride 1, MP: 2×2 max pool, ReLU: Rectified Linear Unit, ConvT: 2×2 transposed convolution with stride 2. The number of features in the expansion layers is double that of contraction layers due to skip connections that form a concatenation with the contraction layers.

No.	Contract	Parameters	No.	Expand	Parameters
1	Conv	$8 \times 3 \times 3 \times 3 + 8$	18	ConvT	$32 \times 64 \times 2 \times 2 + 32$
2	Conv	$8 \times 8 \times 3 \times 3 + 8$	19	Conv	$32 \times 64 \times 3 \times 3 + 32$
3	MP	0	20	ReLU	0
4	Conv	$16 \times 8 \times 3 \times 3 + 16$	21	Conv	$32 \times 32 \times 3 \times 3 + 32$
5	ReLU	0	22	ReLU	0
6	Conv	$16 \times 16 \times 3 \times 3 + 16$	23	ConvT	$16 \times 32 \times 2 \times 2 + 16$
7	ReLU	0	24	Conv	$16 \times 32 \times 3 \times 3 + 16$
8	MP	0	25	ReLU	0
9	Conv	$32 \times 16 \times 3 \times 3 + 32$	26	Conv	$16 \times 16 \times 3 \times 3 + 16$
10	ReLU	0	27	ReLU	0
11	Conv	$32 \times 32 \times 3 \times 3 + 32$	28	ConvT	$8 \times 16 \times 2 \times 2 + 8$
12	ReLU	0	29	Conv	$8 \times 16 \times 3 \times 3 + 8$
13	MP	0	30	ReLU	0
14	Conv	$64 \times 32 \times 3 \times 3 + 64$	31	Conv	$8 \times 8 \times 3 \times 3 + 8$
15	ReLU	0	32	ReLU	0
16	Conv	$64 \times 64 \times 3 \times 3 + 64$	33	Linear	$2 \times 8 + 2$
17	ReLU	0			
				Total	120,834

Accuracy of NFT detections was computed independently for each step of the algorithm. Per-pixel probabilities of tau were evaluated using 10-fold cross validation. Results for each brain sample are summarized in Tables B.2 and B.3.

Table B.2: 10-fold cross validation accuracy statistics for training data of brain sample 1.

Trial	AUC	Precision	Recall	Accuracy
1	0.9997	0.0455	1.0000	0.9928
2	0.9983	0.0938	0.9917	0.9829
3	0.9963	0.1148	0.9846	0.9706
4	0.9984	0.2079	0.9971	0.9877
5	0.9796	0.3010	0.9242	0.9543
6	0.9915	0.0500	0.9620	0.9597
7	0.9865	0.2462	0.9031	0.9899
8	0.9239	0.0081	0.7079	0.9520
9	0.9989	0.1714	1.000	0.9939
10	0.9867	0.0255	0.9406	0.9454
Average	0.9860	0.1264	0.9411	0.9729

Table B.3: 10-fold cross validation accuracy statistics for training data of brain sample 2.

Trial	AUC	Precision	Recall	Accuracy
1	0.9559	0.0234	0.9739	0.8665
2	0.9959	0.0350	0.9668	0.9732
3	0.9947	0.0211	0.9585	0.9733
4	0.9647	0.0341	0.9045	0.9332
5	0.9993	0.2878	0.9967	0.9898
6	0.9979	0.2268	0.9911	0.9756
7	0.9863	0.1502	0.9582	0.9464
8	0.9957	0.1604	0.9805	0.9611
9	0.9915	0.0899	0.9695	0.9688
10	0.9907	0.1108	0.9634	0.9579
Average	0.9873	0.1140	0.9663	0.9546

Segmentations of probability maps into discrete NFTs were validated through direct comparison to manual annotations of NFTs in a subset of annotated image patches reserved for validation. Between 5 and 20 mm^2 patches in the region of the ERC on 10 roughly consecutive slices of one brain were selected for annotation. Per pixel annotations were completed by a single individual over the course of 2-3 weeks. Each patch totaled approximately $2,500,000$ pixels, yielding a total of 25 million for the 10 patches–on

the order of the number of voxels for 20 whole brain MRIs at 1 mm resolution. Total counts of NFTs within each patch yielded through manual annotation versus machine detection were compared 1:1 (see Figure B.1) and demonstrated concordance both in relative trends slice-to-slice and in absolute number of NFTs per patch.

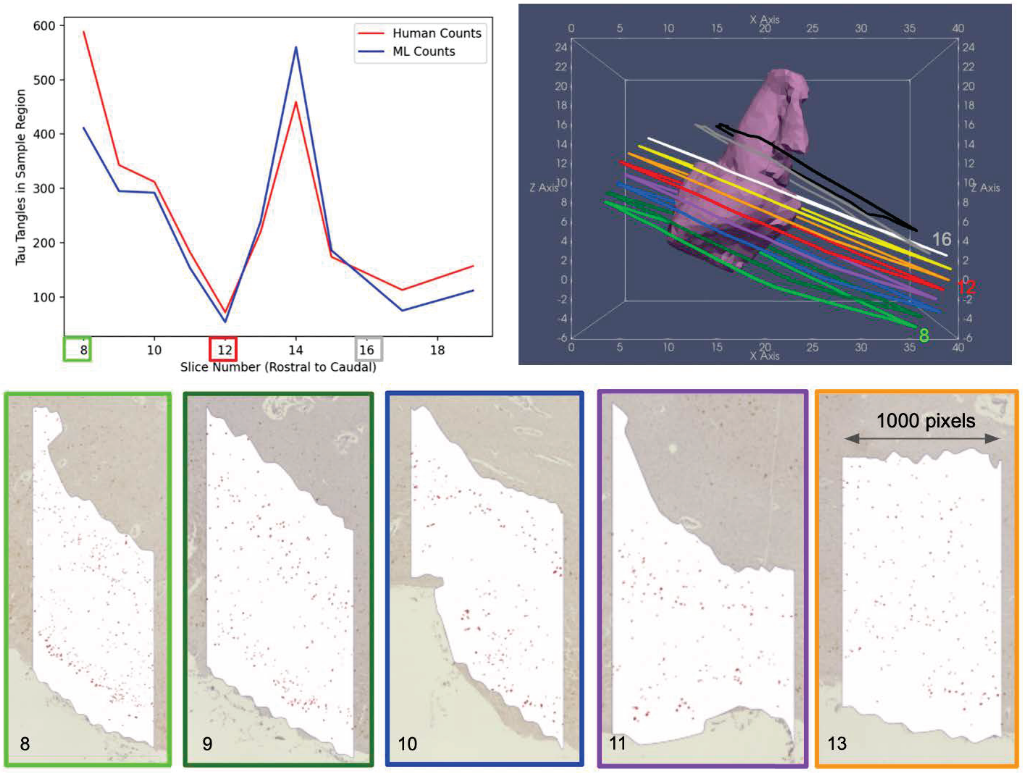

Figure B.1: Selected histology slices with 2D segmentations (top row) ordered left to right as rostral to caudal. Corresponding MRI slices with 3D segmentations mapped to 2D via transformations φ, ϕ_n (second row).Counts of neurofibrillary tau tangles (NFTs) within patches of ERC computed from manual annotations (red) and machine prediction (blue) (third row, left). Outlined sections of histology from which validation set of ERC patches was taken are plotted post transformation in coordinate space of Mai atlas with 3D reconstruction of total ERC (third row, right). Example patches in ERC (white) with NFTs annotated (red) for five slices (bottom).

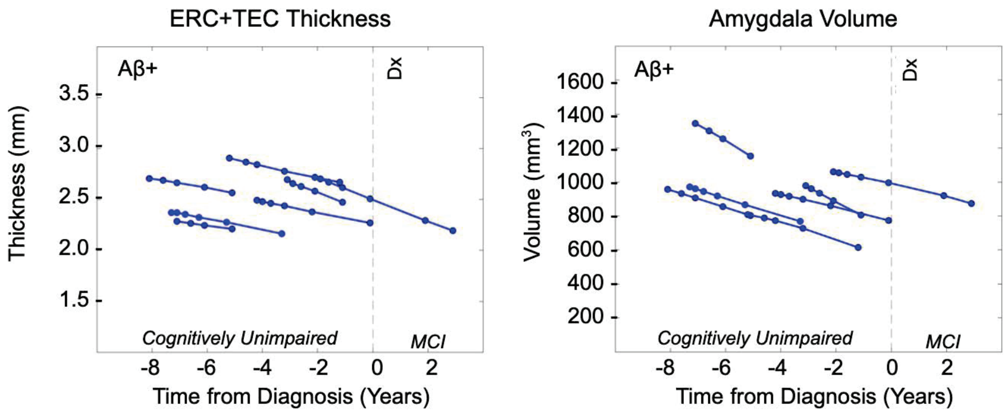

Figure C.1: Estimated atrophy in combined ERC and TEC and amygdala following longitudinal diffeomorphometry in CU $\rightarrow$ MCI subjects with Aβ+ status at baseline. ERC and TEC atrophy measured as percent loss in thickness per year, with mean of 2.52% and standard deviation of 0.98%. Amygdala atrophy measured as percent loss in volume per year, with mean of 5.76% and standard deviation of 1.81%.

Appendix C. Atrophy Rate by Amyloid Status

The CU $\rightarrow$ MCI cohort ($n = 16$) was further delineated into two subsets: those with confirmed Aβ+ status at baseline ($n = 7$) and those without ($n = 9$). Of those without confirmed positive status, 5 subjects did not possess CSF measures and 4 subjects were confirmed with A$\beta-$ status. Aβ status was provided in the ADNI database, with a cutoff of 192 pg/mL. Estimated trajectories of atrophy in combined ERC and TEC thickness and in amygdala volume are shown in Figure C.1 for subjects with confirmed Aβ+ status at baseline.
